# MULTISENSORY LOSS AND DEPRESSION IN THE ATHEROSCLEROSIS RISK IN COMMUNITIES NEUROCOGNITIVE STUDY (ARIC-NCS)

**DOI:** 10.1093/geroni/igae098.4027

**Published:** 2024-12-31

**Authors:** Joseph Shen, Alison Huang, Jennifer Deal, Honglei Chen, Anna Kucharska-Newton

**Affiliations:** Johns Hopkins Bloomberg School of Public Health, Baltimore, Maryland, United States; Johns Hopkins Bloomberg School of Public Health, Baltimore, Maryland, United States; Johns Hopkins University, Baltimore, Maryland, United States; Michigan State University, Michigan State University, Michigan, United States; University of North Carolina at Chapel Hill, Chapel Hill, North Carolina, United States

## Abstract

Multisensory loss is a potentially modifiable risk factor for depression, but population-level evidence is lacking. This study quantified the association between multisensory loss across four senses (hearing, vision, smell, touch) and depressive symptoms in older US adults. Data (N=812) were from the ARIC-NCS and the Eye Determinants of Cognition study joint cohort (2016-2017), an observational study of older adults from Washington County, MD and Jackson, MS. 812 participants with complete data on hearing, vision, olfaction, peripheral neuropathy, depression, and demographic and health covariates were included. Sensory loss was measured using objective tests (pure tone audiometry [hearing], Early Treatment of Diabetic Retinopathy Study chart [vision], Sniffin’ Sticks tests [olfaction], and monofilament tests [peripheral neuropathy]). Multisensory loss was analyzed as a count (0-4). Depressive symptoms were measured by the 11-item Center for Epidemiologic Studies Depression (CES-D-11) scale. Scores were modeled continuously. The incidence rate ratio of depressive symptoms associated with number of sensory losses was assessed using covariate adjusted negative binomial regression. Of 812 participants (aged 71-93 years, 62.7% female, 58.6% White, 43.3% with higher than a high school education) 30.3% had one sensory loss, 31.4% had two, 17.6% had three, and 5.3% had four. Each additional sensory loss was associated with a 10% (Incidence rate ratio of CES-D-11 Score: 1.10; 95% CI: 1.02-1.20) increase in CES-D-11 score in the fully adjusted model. Multisensory loss is associated with depression in older adults. Clinical awareness of this association is valuable for the promotion of mental well-being among patients with sensory loss.

